# Improving the Visualization of the Adrenal Veins Using Virtual Monoenergetic Images from Dual-Energy Computed Tomography before Adrenal Venous Sampling

**DOI:** 10.3390/tomography9020040

**Published:** 2023-02-23

**Authors:** Yu Wang, Xiaohong Chen, Guoxiong Lu, Yun Su, Lingjie Yang, Guangzi Shi, Fang Zhang, Jiayi Zhuo, Xiaohui Duan, Huijun Hu

**Affiliations:** 1 Department of Radiology, Sun Yat-sen Memorial Hospital, Sun Yat-sen University, No. 107 Yanjiang Road West, Guangzhou 510120, China; 2 Guangdong Provincial Key Laboratory of Malignant Tumor Epigenetics and Gene Regulation, Medical Research Center, Sun Yat-sen Memorial Hospital, Sun Yat-sen University, Guangzhou 510120, China

**Keywords:** dual-energy CT, virtual monoenergetic images, adrenal venous sampling, primary aldosteronism

## Abstract

(1) Background: This study explored the optimal energy level in advanced virtual monoenergetic images (VMI+) from dual-energy computed tomography angiography (DE-CTA) for adrenal veins visualization before adrenal venous sampling (AVS). (2) Methods: Thirty-nine patients were included in this prospective single-center study. The CT value, noise, signal-to-noise ratio (SNR) and contrast-to-noise ratio (CNR) were measured in both adrenal veins and abdominal solid organs and were then compared between VMI+ within the range of 40–80 kiloelectron volt (keV). The visualization rate of the adrenal veins and the overall image quality of solid organs were subjectively compared among different keV VMI+. The AVS success rate was recorded for 20 patients. (3) Results: For the adrenal veins, 40 keV VMI+ had the peak CT value, noise and CNR (*p* < 0.05). Subjectively, the visualization rate was the highest at 40 keV (100% for the right adrenal vein, and 97.4% for the left adrenal vein) (*p* < 0.05). For solid organs, the CT value, noise and CNR at 50 keV were lower than those at 40 keV (*p* < 0.05), but the SNR was similar between 40 keV and 50 keV. The overall subjective image quality of solid organs at 50 keV was the best (*p* < 0.05). The AVS success rate was 95%. (4) Conclusions: For VMI+, 40 keV was the preferential energy level to obtain a high visualization rate of the adrenal veins and a high success rate of AVS, while 50 keV was the favorable energy level for the depiction of abdominal organs.

## 1. Introduction

Primary aldosteronism (PA) is the most frequent cause of secondary hypertension in adults, with a prevalence of 1.4%–10% in hypertensive patients [1]. Because PA introduces irreversible damage to the target organs, performing an early diagnosis and applying specific treatments are crucial for reducing the risk of cardiovascular disease events [1,2]. Currently, the main treatment method for unilateral PA is surgery, whereas bilateral PA is pharmacologically treated using the mineralocorticoid receptor antagonist [3]. As a result, selecting the optimal treatment for PA patients necessitates distinguishing between a unilateral aldosterone-producing adenoma and bilateral adrenal hyperplasia.

Adrenal venous sampling (AVS), firstly introduced by Melby et al. [4], has been recommended as the gold standard for certifying the lateralization of aldosterone secretion and provides additional functional information [5]. Unfortunately, the anatomic variation and small size of the adrenal vein increase the technical complexity of the AVS procedure, leading to a variable success rate of AVS for bilateral cannulation, from 42% to 98%, thus limiting its practical applications [6,7]. Thereby, defining both the location and the anatomical variation in the adrenal vein can provide the interventional radiologist with an adrenal vein map before AVS to improve the success rate of catheterization. Currently, contrast-enhanced multi-detector computed tomography (MDCT) is the most commonly used method for the visualization of the adrenal veins prior to AVS [8,9,10]. Previous studies indicated that the visualization rates of the right adrenal vein (RAV) were 93.2% [8], 92.6% [9] and 98% [10] in combination with multi-phase images using the 64-row MDCT system. However, the potential drawbacks of these studies were that the multi-phase dynamic CT protocol requires high radiation doses, and contrast resolution is relatively low in MDCT. Therefore, advanced technology for improving the visualization of the adrenal veins with high contrast resolution before AVS and for reducing the radiation dose simultaneously for computed tomography angiography (CTA) examinations is coming into focus.

Dual-energy computed tomography (DECT) is a newer form of CT technology that allows for simultaneously acquiring datasets at various kiloelectron volt (keV) levels by using a single X-ray tube to distinguish and quantify different material compositions [11]. It allows the generation of various imaging datasets, including virtual non-contrast (VNC) images, virtual monoenergetic images (VMI), material density (MD) images and virtual non-calcium (VNCa) images [12], which can provide several advantages such as material characterization, contrast medium dose and radiation exposure saving, artifacts and noise improvement, and superior resolutions [13]. Traditionally, VMI at arbitrary energies can be synthesized with first-generation conventional monoenergetic reconstruction algorithms [14]. The attenuation in VMI will notably increase when the chosen energy level approximates the K-edge of iodine, but the image noise substantially increases in low-keV VMIs. To overcome this limitation, a novel technique (noise-optimized VMI algorithms, VMI+, i.e., second-generation monoenergetic reconstruction algorithms) was recently introduced by applying a spatial frequency-based technique to combine the information from low-keV (high-contrast property) and high-keV images (low-noise property), which can balance the attenuation and image noise to improve the signal-to-noise ratio (SNR) and contrast-to-noise ratio (CNR) simultaneously [11,15,16]. Previously, VMI+ reconstructions at low keV were applied in CTA to improve the visualization of the small vessels, such as the bronchial arteries [17], coronary arteries [18], pancreatic arteries [19] and intrahepatic veins [20]. Only a preliminary study demonstrated a superior objective and subjective assessment of 40 keV VMI+ in comparison with the standard 120 keV images for the evaluation of the RAV [21]. However, the application of VMI+ at different keV to improve the visualization of the adrenal veins in CTA has not been comprehensively investigated.

The purpose of this study was to evaluate VMI+ reconstructions at different keV to determine the optimal energy level for the best visualization of the adrenal veins on CTA before patients are submitted to AVS.

## 2. Materials and Methods

### 2.1. Patients

The institutional review board approved this prospective single-center study, and informed consent was obtained from each patient. Between January 2022 and August 2022, patients who had hypertension with suspected PA and about to undergo adrenal dual-energy CTA (DE-CTA) examinations on a DECT scanner in Sun Yat-sen Memorial Hospital, Sun Yat-sen University, were included in this study. The following conditions were excluded: pregnancy, severe hepatic and renal dysfunction, allergy to iodinated contrast material, malnutrition, unstable clinical conditions and images with severe motion or shadow artifacts.

### 2.2. CT Examinations

All examinations were performed on a third-generation DECT scanner (SOMATOM Force, Siemens Healthcare, Erlangen, Germany). The scan range was from the top of the adrenal gland to the lower edge of both kidneys in a craniocaudal direction. Arterial phase, venous phase, and excretory phase were performed using two X-ray tubes with different kV tube voltages (tube A: 90 kV; reference current-time product of 400 mAs per rotation; tube B: Sn 150 kV; reference current-time product of 250 mAs per rotation). The used parameters were as follows: gantry rotation of time 1 s; pitch of 0.8; collimation of 128 × 0.6 mm. Automatic exposure control (CAREdose4D, Siemens Healthcare, Erlangen, Germany) was used in all studies. The images were reconstructed at 1 mm thickness and 1 mm intervals using a reconstruction kernel of Br36. After the acquisition of non-contrast images, a non-ionic contrast medium (Ioversol, 350 mgI/mL, Hengrui Pharmaceutical Co, Ltd., Jiangsu, China) was injected at a flow rate of 4 mL/s and a dose of 1.5 mL/kg through the median cubital vein by using a power injector (Medrad Stellant, Siemens Healthcare, Erlangen, Germany) followed by a 30 mL saline chaser. The region of interest (ROI) was placed on the descending aorta at the diaphragm level, and the start of the arterial phase scanning operation was automatically launched 10 s after the attenuation reached the pre-defined threshold of 100 HU using the bolus-tracking technique. The venous phase scanning and excretory phase scanning were carried out approximately 20 s and 65 s after the end of the arterial phase, respectively.

### 2.3. Image Reconstruction

The reconstructed CT image data were post-processed on a workstation (Syngo.via, Siemens Healthcare, Erlangen, Germany). VMI+ reconstructions with a 1 mm thickness and a 1 mm increment were obtained from 40 to 80 keV, with 10 keV intervals. Volume-rendered (VR) and maximum-intensity projection (MIP) reconstructions were applied to observe the adrenal veins.

### 2.4. DE-CTA Objective Image Analysis

All image analyses were performed on a dedicated DECT workstation (Syngo.via, Siemens Healthcare, Erlangen, Germany). Objective measurements were performed by a radiologist (G.S.) with 7 years of experience in abdominal CTA.

For the VMI+ analysis, we compared the CT value, noise, SNR and CNR of the adrenal veins and abdominal solid organs in VMI+ reconstructions from 40 to 80 keV by placing ROIs on the target areas. For the adrenal veins, the ROIs were placed in a representative area of the adrenal veins and psoas major muscle to measure the CT value and noise. The SNR and CNR of the adrenal veins were obtained according to the following formulas provided by a prior study [21]: SNR = HU adrenal vein/SD adrenal vein, CNR = (HU adrenal vein − HU muscle)/SD muscle. For the abdominal organs, the ROIs were placed in the liver, spleen, pancreas, kidneys, and adrenal glands to measure the CT value and noise. The SNR and CNR of the solid organs were calculated using the following formulas: SNR = HU organ/SD organ and CNR = (HU organ − HU muscle)/SD muscle [22]. The ROI size and location were as similar as possible to ensure the comparability of the objective values, and all measurements were performed on the cross-sectional images three times and then averaged.

### 2.5. DECT Subjective Image Analysis

Two independent radiologists with 7 (F.Z.) and 10 (X.D.) years of experience independently assessed the images by using a double-blind method. The reviewers were blinded to the energy levels of the VMI+. The images were arranged in the standard window settings, but the reviewers were explicitly allowed to freely alter the window settings. Ratings were determined independently, and series were rated in random order. The final score was decided by consensus if there were disagreements between the 2 readers.

We assessed the degree of visualization in the adrenal veins in VMI+/MIP reconstructions based on a previous study [8]. A four-point scale was applied for the assessment of the degree of visualization in adrenal veins, with the following scores: 4 (excellent visualization; the adrenal vein is clearly visualized, and the vessel is obviously strengthened compared with the surrounding structures), 3 (good visualization; although the contrast of the adrenal vein is not strong, the adrenal vein can be detected accurately), 2 (fair visualization; the adrenal vein has minimal contrast compared with the surrounding structures and is equivocally detected) and 1 (poor visualization; the adrenal vein is not visualized). The scores of 4 and 3 were considered as allowing the visualization of the adrenal vein, and the visualization rate of the adrenal vein was further calculated.

The overall image quality of solid organs in the VMI+ reconstructions was assessed using a 5-point Likert scale, with 5 = excellent, no obvious noise and artifacts and satisfactory anatomical structures and details; 4 = good, slight noise and artifacts and less clear anatomical structure and details; 3 = fair, moderate noise and artifacts and acceptable enhancement, but insufficient for diagnosis; 2 = poor, severe noise and artifacts and inadequate for diagnosis; and 1 = no diagnosis possible [23].

### 2.6. Statistical Analysis

Statistical analysis was performed using SPSS version 25.0 ﻿(IBM Corp., Armonk, NY, USA). The continuous variables were presented as the mean ± SD, and image quality scores were expressed as the median (interquartile range). The distribution of data and the homogeneity of variances were tested by using the Shapiro–Wilk test and the Levene’s test, respectively. The non-parametric Friedman test with post-hoc tests was used to compare the measurements (CT value, noise, CNR, SNR) and image quality of the adrenal veins and abdominal solid organs between multiple groups (40–80 keV VMI+). The interobserver agreement was analyzed by Cohen’s kappa values, which were interpreted as indicating slight agreement (<0.20), fair agreement (0.21–0.40), moderate agreement (0.41–0.60), substantial agreement (0.61–0.80) and perfect agreement (0.81–1.00). A *p* < 0.05 was considered statistically significant for all the above tests.

## 3. Results

### 3.1. Participant Characteristics

**Figure 1:**
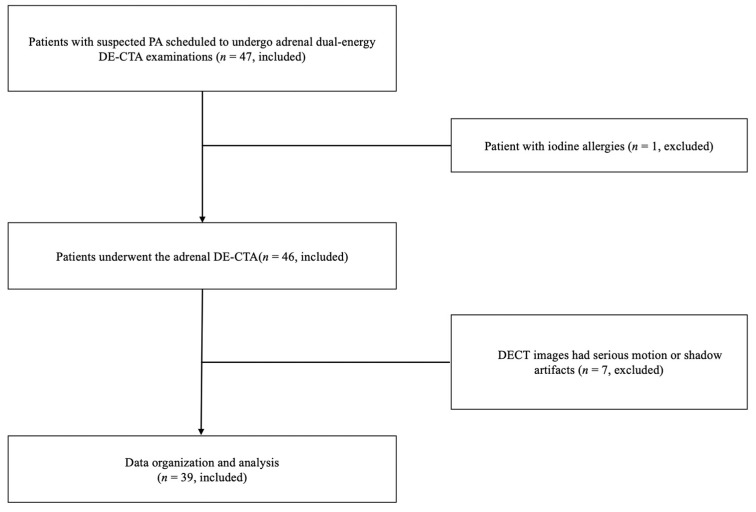
Flow diagram for subject selection. PA, primary aldosteronism; DE-CTA, dual energy-computed tomography angiography; DECT, dual-energy computed tomography.

Of the 47 patients initially included in our study, 8 participants were excluded because of iodine allergies (*n* = 1) and serious motion or shadow artifacts on the DECT images (*n* = 7). The patient enrollment flow is shown in Figure 1.

Finally, 39 patients in an age range from 24.0 to 48.0 years were included in this study. The baseline characteristics of the included patients are summarized in Table 1.

### 3.2. Objective Analysis of VMI+ Series from Venous Phase Data

For the adrenal veins, there were gradually decreasing trends in CT value, noise and CNR from the 40 keV to the 80 keV VMI+ reconstructions. A significantly higher CT value was found for the 40 keV VMI+ than for the 50–80 keV ones of the adrenal veins (all *p* < 0.05). The CT value at 40 keV VMI+ increased approximately by 23%, 50%, 62% and 69% compared to the value at 50–80 keV for the right adrenal vein and approximately by 31%, 50%, 62% and 71% for the left adrenal vein. For the adrenal veins, the image’s noise decreased in the 50–80 keV VMI+ compared to the 40 keV images, with statistical significance (all *p* < 0.05). The CNR values of the 40 keV VMI+ of the adrenal veins were significantly higher in comparison with those of the 50–80 keV VMI+ (all *p* < 0.05) and increased approximately by 12%, 25%, 37% and 42% compared to those of the 50–80 keV images for the right adrenal vein and by 12%, 25%, 38% and 49% for the left adrenal vein. For the SNR, no significant difference was observed between the 40 and the 50 keV VMI+ reconstructions (*p* > 0.05).

In the case of the kidneys, adrenal glands, liver, spleen and pancreas, the CT value, noise and CNR at 40 keV were higher than those at 50 keV VMI+ (all *p* < 0.05), but there was no statistical difference for the SNR between 40 and 50 keV VMI+ (*p* > 0.05), except for the SNR for the liver (*p* = 0.02). The VMI+ reconstructions at 50 keV showed higher CT value, noise and CNR compared with the 60–80 keV images (all *p* < 0.05). There was no statistical difference in SNR between 50 and 60 keV VMI+ (all *p* > 0.05), except for the SNR for the liver and pancreas (*p* < 0.05). The VMI+ at 50 keV reflected a higher SNR than the 70 keV and 80 keV images for the left kidney, adrenal glands and liver (all *p* < 0.05). The results of the objective analysis of the adrenal veins and abdominal solid organs are shown in Table 2.

### 3.3. Subjective Analysis of VMI+ Series from Venous Phase Data

**Figure 2:**
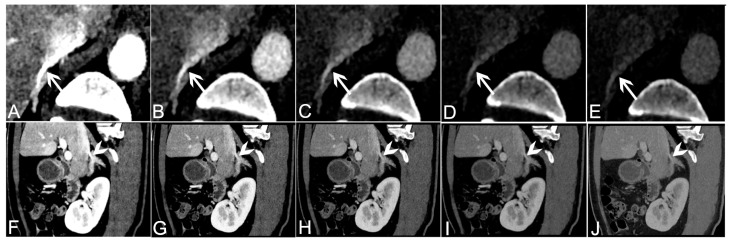
A 61-year-old male patient with a right adrenal adenoma underwent DE-CTA. Axial VMI+ reconstructions at a level of 40 keV (**A**), 50 keV (**B**), 60 keV (**C**), 70 keV (**D**) and 80 keV (**E**). The right adrenal vein (arrows) can be visualized. In this case, the right adrenal vein was filled with the contrast material at 40 keV. The CT value and CNR of the right adrenal vein at 40 keV were higher than those of the 50–80 keV VMI+ reconstructions. Sagittal images were reconstructed to visualize the right adrenal vein (arrowheads): 40 keV (**F**), 50 keV (**G**), 60 keV (**H**), 70 keV (**I**) and 80 keV (**J**). The clarity and observation of solid organs in 50 keV VMI+ reconstructions were better than those in other keV reconstructions. DE-CTA, dual-energy computed tomography angiography; VMI+, advanced virtual monoenergetic images; keV, kiloelectron volt; CNR, contrast-to-noise ratio.

**Figure 3:**
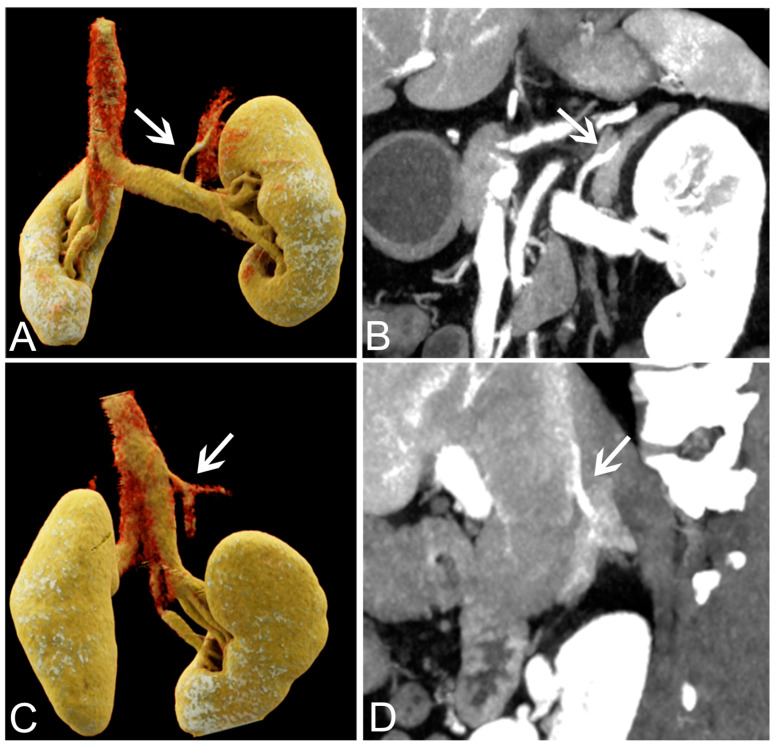
VR and MIP reconstructions of the adrenal veins in dual-energy computed tomography images. VR (**A**) and MIP (**B**) images of the left adrenal vein (white arrows) in a 42-year-old female patient with a left adrenal adenoma. VR (**C**) and MIP (**D**) images of the right adrenal vein (white arrows) in a 42-year-old female patient with a right adrenal adenoma. VR, volume-rendered; MIP, maximum-intensity projection.

We defined a score of 4 or 3 as that allowing the visualization of the adrenal veins. The best visualization rate of the adrenal veins was obtained for the 40 keV VMI+. For the right adrenal vein, the overall detectability was 100%, 87.18%, 46.15%, 10.26% and 0% in 40–80 keV VMI+. For the left adrenal vein, the overall detectability was 97.44%, 94.87%, 64.11%, 25.64% and 0% in 40–80 keV VMI+. The 40 keV VMI+ reconstructions obtained superior scores compared to the other VMI+ series, with significant differences (all *p* < 0.05). There was a good agreement in the visualization scores between the two radiologists for the 40–80 keV VMI+ (κ from 0.78 to 0.89). The visualization scores and detectability of the adrenal veins are presented in Table 3, and images are shown in Figure 2. Representative MIP and VR images are shown in Figure 3.

The subjective overall image quality scores of solid organs were determined best in the 50 keV VMI+ reconstructions with high interrater agreement, followed by the 40 keV and the 60 keV reconstructions, with significant differences (all *p* < 0.001). The VMI+ reconstructions at 70 keV and 80 keV received lower scores compared with the 50 keV images (all *p* < 0.001) (Table 4).

### 3.4. Success Rate of AVS

AVS was performed by an experienced radiologist after the evaluation of the adrenal veins position and anatomical variants in the DECT images. Finally, a total of 20 patients underwent AVS, with a success rate of 95% (19/20).

## 4. Discussions

In the present study, we explored the optimal energy level of VMI+ for the best visualization of the adrenal veins on DE-CTA for patients planned to undergo AVS. Our results showed that the optimal keV level was 40 keV for the visualization of the adrenal veins in adrenal DE-CTA prior to AVS, based on objective and subjective evaluations. However, the VMI+ reconstruction at 50 keV was the favorable images for the depiction of abdominal organs based on a subjective evaluation of image quality.

DECT is a relatively newer form of CT technique to improve the visualization of enhanced vessels without increasing the doses of radiation and contrast agent [19]. To achieve an excellent vascular visualization, increasing vascular attenuation with a low image noise is the main goal in DE-CTA. Typically, the observation of the enhanced vessels primarily depends on the degree of vascular contrast, which can make vessels appear artifactually bright compared to the surrounding structures. On the contrary, image noise and streak artifacts will contribute to a poor image quality, resulting in a poor observation of vessels in low-keV images. Therefore, the optimal image quality must be selected carefully to balance vessel contrast and image noise, and CNR is the major objective indicator of vascular enhancement [19,24]. Some previous studies [24,25] indicated that a greater visualization of vessels and an increased diagnostic confidence could be obtained using VMI images in DE-CTA. VMI images ranging from 60 to 70 keV were recommended for achieving the best overall image quality due to the severe noise at lower energy levels. In our study, we compared the image quality objectively and subjectively among various energy levels to identify the favorable energy level for the best visualization of the adrenal veins before AVS by using the VMI+ algorithm. In the presented results, the objective analysis of VMI+ reconstructions showed the peak CT value and CNR values at 40 keV. The mild increase in image noise at 40 keV was overcome by the substantially increased attenuation. Consequently, an increased CNR could be observed in the 40 keV images in comparison to the other keV images. Moreover, the objective results were consistent with the subjective findings. The visualization rate reached 100% (39/39) for the right adrenal vein and 97.43% (38/39) for the left adrenal vein, allowing the planning of AVS using 40 keV VMI+ reconstructions. A prior study [8] showed that the visualization rates of the RAV were 93.2% in MDCT and 84.2% in MR imaging, and by combining the results of MDCT and MR, the overall visualization rate of the RAV reached 96.8%. Our results indicated a significantly better visualization rate than those reported. Based on this, the success rate of AVS achieved 95.0% when performed with the DECT images for the evaluation of the adrenal vein’s position and anatomical variants in our study. Overall, our results emphasize that the VMI+ reconstructions at 40 keV should be preferred over other energy-level reconstructions for adrenal veins visualization in patients undergoing adrenal DE-CTA. This result is different from those of several previous studies [24,25] but it is similar to those of some recent studies for the detection of coronary arteries [18], bronchial arteries [17] and intrahepatic veins [20] in DE-CTA. These inconsistencies may mainly be due to the different DECT scanners employed. The third-generation DECT used in this study could apply the novel VMI+ algorithm to combine the information on low-keV images (which provide a high iodine attenuation) and high keV images (which provide the optimal noise properties), ultimately allowing for an increased CNR at low keV levels. Taken together, our study demonstrated that the VMI+ technique can overcome noise-based limitations at 40 keV to improve image quality quantitatively and qualitatively for vasculature visualization.

On the other hand, in our study, the subjective overall image quality (incorporating image noise, blurring, and artifacts) of the abdominal solid organs in the 50 keV reconstruction was the best, and the SNR was similar at 40 keV and 50 keV, although the mean CT value and CNR in 50 keV VMI+ reconstructions were lower than those at 40 keV, indicating that the 50 keV reconstructions were the most favorable images for observing the main abdominal solid organs. Considering all these results, we tend to prefer the 40 keV VMI+ reconstructions for the visualization of the adrenal veins before AVS and the 50 keV images for the depiction of abdominal organs. Previously, Kim et al. [22] suggested that the optimal energy level for SNR and CNR was at 40 keV, whereas the optimal energy levels for image quality was at 60 keV in pediatric abdominopelvic DE-CTA. Other investigators also found a similar discrepancy between objective and subjective indicators when evaluating low-keV VMI+ in DE-CTA [26,27,28]. This indicates that the optimal energy level for VMI+ reconstructions may differ depending on whether vascular assessment or the characterization of lesions is performed. The vascular assessment with the highest attenuation and CNR derived from the 40 keV reconstructions in DE-CTA could achieve the best visual effect after VR and MIP reconstructions, even with the largest image noise. In contrast, for the visualization of lesions and abdominal organs with moderate or low mean attenuation and CNR, it was necessary to reduce the influence of noise to achieve better lesion detection and delineation; thus, in this case, the energy level at 50 keV was the optimal choice.

There are several limitations in the present study. First, this study was a single-center study with only 39 patients included. Future studies with larger population samples are required. Second, a lesion’s detectability and delineation in VMI+ at different energy levels were not evaluated in our study because only a proportion of our patients had a detectable lesion. Future studies are necessary for exploring the optimal energy levels of VMI+ for lesion detection and delineation in patients with PA. Last, we calculated the success rate of AVS, although not all patients underwent AVS. A larger sample size for calculating the success rate of AVS is necessary in future studies.

## 5. Conclusions

Our results demonstrated that 40 keV was the preferential energy level for VMI+ reconstructions for the visualization of the adrenal veins to obtain a high success rate of AVS, but 50 keV was the favorable energy level for the depiction of abdominal organs and lesions.

## Figures and Tables

**Table 1:** Demographic and clinical characteristics of the patients.

Patient Characteristics	Value (%)
Age (years old)	45.08 ± 11.98
Sex	
male	*n* = 19 (48.71)
female	*n* = 20 (51.29)
BMI (kg/m^2^)	24.90 ± 3.41

Continuous variables are described as mean ± SD, and categorical variables are presented as a number (percentage). BMI, body mass index.

**Table 2:** CT value, noise, SNR and CNR for the adrenal veins and main abdominal organs in different VMI+ images.

	40 keV	50 keV	60 keV	70 keV	80 keV	*p* ^a^	*p* ^b^			
							*p* 40 vs. 50	*p* 40 vs. 60	*p* 40 vs. 70	*p* 40 vs. 80
Right adrenal vein										
CT value	489.72 ± 119.72	377.23 ± 79.42	244.49 ± 55.15	187.30 ± 40.44	150.63 ± 31.29	<0.001 *	0.005 *	<0.001 *	<0.001 *	<0.001 *
noise	31.60 ± 8.81	21.95 ± 5.86	16.11 ± 4.14	12.54 ± 3.17	11.62 ± 3.58	<0.001 *	0.005 *	<0.001 *	<0.001 *	<0.001 *
CNR	14.82 ± 5.39	13.05 ± 4.78	11.16 ± 4.09	9.36 ± 3.54	8.57 ± 1.06	<0.001 *	0.005 *	<0.001 *	<0.001 *	<0.001 *
SNR	16.18 ± 4.88	16.02 ± 4.73	15.83 ± 4.64	15.62 ± 4.62	14.39 ± 6.32	<0.001 *	1	0.88	0.28	<0.001 *
Left adrenal vein										
CT value	528.05 ± 98.26	362.44 ± 65.69	261.72 ± 46.34	199.60 ± 34.91	150.63 ± 31.29	<0.001 *	0.005 *	<0.001 *	<0.001 *	<0.001 *
noise	33.95 ± 9.04	23.78 ± 6.06	17.65 ± 4.28	13.91 ± 3.22	11.55 ± 2.60	<0.001 *	0.005 *	<0.001 *	<0.001 *	<0.001 *
CNR	17.42 ± 5.08	15.27 ± 4.62	12.98 ± 4.06	10.77 ± 3.49	8.83 ± 3.02	<0.001 *	0.005 *	<0.001 *	<0.001 *	<0.001 *
SNR	16.48 ± 4.89	16.08 ± 4.71	15.59 ± 4.53	15.03 ± 4.40	14.51 ± 4.33	0.253	0.053	<0.001 *	<0.001 *	<0.001 *
							*p* ^c^			
							*p* 50 vs. 40	*p* 50 vs. 60	*p* 50 vs.70	*p* 50 vs.80
Right adrenal gland										
CT value	331.10 ± 88.59	227.49 ± 59.53	164.48 ± 42.02	125.62 ± 31.39	100.71 ± 24.74	<0.001 *	0.005 *	0.005 *	<0.001 *	<0.001 *
noise	28.45 ± 5.48	20.46 ± 3.83	15.69 ± 2.88	12.82 ± 2.35	11.03 ± 2.06	<0.001 *	0.005 *	0.005 *	<0.001 *	<0.001 *
CNR	8.95 ± 3.85	7.55 ± 3.42	6.08 ± 2.98	4.67 ± 2.61	1.94 ± 1.32	<0.001 *	0.005 *	0.005 *	0.001 *	<0.001 *
SNR	11.87 ± 3.15	11.37 ± 3.09	10.74 ± 2.99	10.06 ± 2.84	9.39 ± 2.68	<0.001 *	0.012	0.012	<0.001 *	<0.001 *
Left adrenal gland										
CT value	324.22 ± 101.86	220.39 ± 68.26	157.71 ± 48.32	119.06 ± 36.20	94.28 ± 28.60	<0.001 *	0.005 *	0.005 *	<0.001 *	<0.001 *
noise	31.51 ± 5.31	22.70 ± 3.88	17.40 ± 3.12	14.20 ± 2.75	12.18 ± 2.57	<0.001 *	0.005 *	0.005 *	<0.001 *	<0.001 *
CNR	9.25 ± 4.08	7.55 ± 3.42	5.84 ± 2.92	4.22 ± 2.51	2.83 ± 2.21	<0.001 *	0.005 *	0.005 *	<0.001 *	<0.001 *
SNR	10.51 ± 3.61	9.95 ± 3.44	9.33 ± 3.29	8.69 ± 3.13	8.07 ± 2.98	<0.001 *	0.081	0.081	<0.001 *	<0.001 *
Right kidney										
CT value	240.82 ± 59.67	167.23 ± 39.53	122.47 ± 27.40	94.87 ± 20.06	77.18 ± 15.49	<0.001 *	0.005 *	0.005 *	<0.001 *	<0.001 *
noise	31.44 ± 4.66	21.91 ± 3.33	16.17 ± 2.61	12.61 ± 2.23	10.52 ± 2.02	<0.001 *	0.005 *	0.005 *	<0.001 *	<0.001 *
CNR	19.48 ± 7.51	12.83 ± 6.56	14.41 ± 5.84	11.9 ± 5.05	5.46 ± 2.36	<0.001 *	0.005 *	0.005 *	<0.001 *	<0.001 *
SNR	7.76 ± 2.02	7.75 ± 1.98	7.72 ± 1.98	7.66 ± 2.02	7.58 ± 2.10	0.023	0.72	0.56	0.13	<0.01 *
Left kidney										
CT value	255.30 ± 60.60	176.41 ± 40.23	128.43 ± 27.98	98.84 ± 20.57	79.88 ± 15.98	<0.001 *	0.006 *	0.006 *	<0.001 *	<0.001 *
noise	29.75 ± 5.38	20.65 ± 3.72	15.17 ± 2.77	12.42 ± 1.39	10.46 ± 1.22	<0.001 *	0.006 *	0.006 *	<0.001 *	<0.001 *
CNR	20.23 ± 7.71	17.53 ± 6.73	15.13 ± 5.04	11.92 ± 4.83	9.52 ± 4.03	<0.001 *	0.006 *	0.006 *	<0.001 *	<0.001 *
SNR	8.89 ± 2.75	8.86 ± 2.67	8.79 ± 2.62	8.68 ± 2.69	8.54 ± 2.63	<0.001 *	0.51	0.38	0.03 *	0.005 *
Liver										
CT value	232.86 ± 42.20	182.31 ± 39.80	141.17 ± 28.70	115.81 ± 22.09	99.55 ± 18.02	<0.001 *	0.005 *	0.005 *	<0.001 *	<0.001 *
noise	29.87 ± 3.34	20.93 ± 2.30	15.60 ± 1.71	12.42 ± 1.39	10.46 ± 1.22	<0.001 *	0.005 *	0.005 *	<0.001 *	<0.001 *
CNR	5.87 ± 2.43	5.23 ± 2.19	4.54 ± 1.97	3.89 ± 1.81	1.91 ± 1.12	<0.001 *	0.008 *	0.01 *	<0.001 *	<0.001 *
SNR	8.45 ± 2.06	8.81 ± 2.13	9.18 ± 2.22	9.48 ± 2.32	9.71 ± 2.44	<0.001 *	0.02 *	0.03 *	<0.001 *	<0.001 *
Spleen										
CT value	381.84 ± 78.21	266.61 ± 51.41	196.52 ± 35.14	153.30 ± 25.15	125.59 ± 18.79	<0.001 *	0.005 *	0.005 *	<0.001 *	<0.001 *
noise	28.73 ± 4.46	19.95 ± 2.87	14.71 ± 2.01	11.56 ± 1.55	9.61 ± 1.33	<0.001 *	0.005 *	0.005 *	<0.001 *	<0.001 *
CNR	11.52 ± 3.4	10.02 ± 2.91	8.45 ± 2.44	6.96 ± 2.03	5.65 ± 1.71	<0.001 *	0.005 *	0.005 *	<0.001 *	<0.001 *
SNR	13.52 ± 3.02	13.59 ± 3.01	13.61 ± 3.01	13.54 ± 3.02	13.39 ± 3.07	0.62				
Pancreas										
CT value	279.40 ± 61.93	198.23 ± 40.64	148.81 ± 27.85	118.33 ± 20.16	98.79 ± 15.43	<0.001 *	0.005 *	0.005 *	<0.001 *	<0.001 *
noise	29.12 ± 4.00	20.63 ± 2.64	15.57 ± 1.92	12.56 ± 1.55	10.17 ± 1.77	<0.001 *	0.005 *	0.005 *	<0.001 *	<0.001 *
CNR	7.52 ± 4.46	6.43 ± 2.3	5.3 ± 1.91	4.23 ± 1.59	3.30 ± 1.36	<0.001 *	0.005 *	0.005 *	<0.001 *	<0.001 *
SNR	9.71 ± 2.28	9.74 ± 2.26	7.50 ± 2.11	9.61 ± 2.37	10.13 ± 3.61	<0.001 *	1	<0.001 *	0.25	0.77

Values are given as mean ± SD. *p* ^a^ is the comparison among multiple groups. *p* ^b^ is the comparison between the 40 keV and the other energy level VMI+. *p* ^c^ is the comparison between the 50 keV and the other energy level VMI+. * Significant difference (*p* < 0.05). VMI+, advanced virtual monoenergetic images; keV, kiloelectron volt; SNR, signal-to-noise ratio; CNR, contrast-to-noise ratio.

**Table 3:** Visualization score and detectability of the adrenal veins in different VMI+.

	40 keV VMI+	50 keV VMI+	60 keV VMI+	70 keV VMI+	80 keV VMI+
Right Adrenal Vein					
4 (excellent)	26	3	3	0	0
3 (good)	13	31	15	4	0
2 (fair)	0	5	21	6	7
1 (poor)	0	0	0	29	32
detectability	100% (39/39)	87.18% (34/39)	46.15% (18/39)	10.26% (4/39)	0% (0/39)
score	4 (4,4)	3 (3,3)	2 (2,3)	2 (2,2)	1 (1,1)
*p*		<0.01 *	<0.001 *	<0.001 *	<0.001 *
kappa	0.80	0.86	0.86	0.73	0.80
Left adrenal vein					
4 (excellent)	24	7	0	0	0
3 (good)	14	30	25	10	0
2 (fair)	1	2	14	26	20
1 (poor)	0	0	0	3	19
detectability	97.44% (38/39)	94.87% (37/39)	64.11% (25/39)	25.64% (10/39)	0% (0/39)
score	4 (4,4)	3 (3,3)	3 (2,3)	2 (2,3)	2 (1,2)
*p*		0.008 *	<0.001 *	<0.001 *	<0.001 *
kappa	0.79	0.87	0.89	0.78	0.89

Scores are medians with interquartile ranges in parentheses. *p* values correspond to the comparison between the 40 keV images and the other energy level VMI+. * Significant difference (*p* < 0.05). VMI+, advanced virtual monoenergetic images; keV, kiloelectron volt.

**Table 4:** Subjective analysis of the main abdominal organs in different VMI+.

	Image Quality	*p*	Kappa
40 keV VMI+	3 (3,4)	<0.001 *	0.86
50 keV VMI+	5 (5,5)		0.72
60 keV VMI+	3 (3,3)	<0.001 *	0.72
70 keV VMI+	2 (2,3)	<0.001 *	0.77
80 keV VMI+	2 (1,2)	<0.001 *	0.85

Data are the median with interquartile ranges in parentheses; *p* values are the results of the comparisons between 50 keV and other keV VMI+. * Significant difference (*p* < 0.05). VMI+, advanced virtual monoenergetic images; keV, kiloelectron volt.

## Data Availability

The data presented in this study are available on request from the corresponding author.
